# QTL fine mapping with Bayes C(π): a simulation study

**DOI:** 10.1186/1297-9686-45-19

**Published:** 2013-06-19

**Authors:** Irene van den Berg, Sébastien Fritz, Didier Boichard

**Affiliations:** 1 INRA, UMR1313 Génétique animale et biologie intégrative, Domaine de Vilvert, 78350 Jouy-en-Josas, France; 2 AGROPARISTECH, UMR1313 Génétique animale et biologie intégrative, 16 rue Claude Bernard, 75231 Paris 05, France; 3 Department of Molecular Biology and Genetics, Faculty of Science and Technology, Aarhus University, DK-8830 Tjele, Denmark; 4 UNCEIA, 149 rue de Bercy, 75012 Paris, France

## Abstract

**Background:**

Accurate QTL mapping is a prerequisite in the search for causative mutations. Bayesian genomic selection models that analyse many markers simultaneously should provide more accurate QTL detection results than single-marker models. Our objectives were to (a) evaluate by simulation the influence of heritability, number of QTL and number of records on the accuracy of QTL mapping with Bayes Cπ and Bayes C; (b) estimate the QTL status (homozygous vs. heterozygous) of the individuals analysed. This study focussed on the ten largest detected QTL, assuming they are candidates for further characterization.

**Methods:**

Our simulations were based on a true dairy cattle population genotyped for 38 277 phased markers. Some of these markers were considered biallelic QTL and used to generate corresponding phenotypes. Different numbers of records (4387 and 1500), heritability values (0.1, 0.4 and 0.7) and numbers of QTL (10, 100 and 1000) were studied. QTL detection was based on the posterior inclusion probability for individual markers, or on the sum of the posterior inclusion probabilities for consecutive markers, estimated using Bayes C or Bayes Cπ. The QTL status of the individuals was derived from the contrast between the sums of the SNP allelic effects of their chromosomal segments.

**Results:**

The proportion of markers with null effect (π) frequently did not reach convergence, leading to poor results for Bayes Cπ in QTL detection. Fixing π led to better results. Detection of the largest QTL was most accurate for medium to high heritability, for low to moderate numbers of QTL, and with a large number of records. The QTL status was accurately inferred when the distribution of the contrast between chromosomal segment effects was bimodal.

**Conclusions:**

QTL detection is feasible with Bayes C. For QTL detection, it is recommended to use a large dataset and to focus on highly heritable traits and on the largest QTL. QTL statuses were inferred based on the distribution of the contrast between chromosomal segment effects.

## Background

The first step in the identification of causative mutations underlying quantitative traits is to search for genomic regions associated with these traits, called quantitative trait loci (QTL). With the availability of genetic markers, a large number of QTL has been reported for various species and traits [1-3]. However, taking the final step from QTL to causative mutation has proven more difficult. In farm animals, most economically important traits are complex and involve a large number of genes, each with a small effect. Furthermore, due to long range linkage disequilibrium, as is the case in dairy cattle [4], several markers can be in linkage disequilibrium with the causative mutation rather than only the markers closest to the mutation. As a consequence, reported QTL often have large confidence intervals [5], making the search for causative mutations difficult, and mutations underlying such traits have been identified only in a few cases [6]. Accurate and precise QTL mapping is thus a prerequisite to the search for causative mutations.

A large variety of models for QTL detection exists, including single-marker models, interval mapping [7] and Bayesian models [8]. Since quantitative traits are likely to be influenced by a large number of QTL, models that analyse all markers simultaneously should give more accurate results than models that analyse only one or a few markers at a time. With the Bayesian models developed for genomic selection, a large number of markers can be analysed simultaneously [9]. In a simulation study by Sahana et al. [10], Bayesian QTL mapping resulted in higher power and more precise QTL locations than single-marker, haplotype-based or mixed-model approaches. When dense single nucleotide polymorphism (SNP) chips are used, loci influencing a trait are assumed to be in linkage disequilibrium with one or more markers. Genomic selection models exploit this linkage disequilibrium to estimate the effects of the markers and genomic breeding values [9]. Although the focus in the development of these models has been to estimate breeding values rather than to identify QTL, the estimated marker effects show potential use for QTL mapping [11]. For the original models proposed by Meuwissen et al. [9], either all markers were included in the model (Bayes A), or the proportion of markers included in the model was fixed at a certain value (Bayes B). To overcome the statistical problems associated with these models reported by Gianola et al. [12], Habier et al. [13] proposed several modifications of the original models, including Bayes Cπ and Bayes C. In Bayes Cπ, the proportion of markers included in the model is assigned a prior distribution and estimated during the analysis, while in Bayes C, the proportion is given a fixed value. In a simulation by Sun et al. [14], Bayes Cπ was successful in identifying large QTL. Bayes C has been used to identify QTL for various traits in beef cattle [15,16], the horse [17] and pigs [18,19].

The performance of the Bayesian genomic selection models is known to be influenced by the genetic architecture of the trait and the number of records used to estimate the marker effects; the accuracy of predicted breeding values decreases with decreasing heritability [20,21] and an increasing number of QTL [21,22] and a large number of records is needed to obtain high accuracy [4]. Although several studies have evaluated the impact of these factors on the accuracy of breeding value estimation, their impact on the use of these models for QTL mapping is unknown.

The regions detected by QTL mapping generally contain a large number of polymorphisms that could be the causative mutation. However, assuming only one true causative mutation in a QTL, an individual’s genotype for the causative mutation should be in concordance with its status for the QTL: if the individual is homozygous for the QTL, it should be homozygous for the causative mutation, and if the individual is heterozygous for the QTL, it should also be heterozygous for the causative mutation [23]. By comparing the status of the QTL and polymorphisms in the QTL region, all polymorphisms with statuses that are not in concordance with the statuses of the QTL can be eliminated as potential causative mutations. Using this approach, Karlsson et al. [24] were able to eliminate more than half of the candidate polymorphisms for a causative mutation involved in coat colour in dogs. However, if non-concordant polymorphisms are eliminated, it is important that prediction of the QTL status is correct. One possibility to determine the QTL status is to compare the estimated marker effects of the two haplotypes that an individual carries in the QTL region: if the effects of the haplotypes are very different, the individual is likely heterozygous for the QTL, while if the difference is close to zero, it is likely to be homozygous.

The first objective of this study was to evaluate by simulation the influence of heritability, number of QTL and number of records on the accuracy of QTL mapping with genomic prediction methods Bayes Cπ and Bayes C. Our second objective was to estimate the accuracy of the prediction of the status of the causative mutation using the estimated marker effects from Bayes C. The study focussed on the largest QTL because they are the natural candidates for molecular identification of a causative mutation. It aimed at answering the following questions: are we able to detect the largest QTL? Are they true QTL? Are we able to infer correctly the QTL status of the individuals?

## Methods

### Simulation

QTL and phenotypes were simulated for different heritabilities and numbers of QTL. An existing real dataset was used as a base for the simulation. It consisted of 4387 genotyped French Holstein bulls, with a pedigree of 12 142 individuals. The genotypes were obtained with the Illumina Bovine SNP50 Bead Chip® by Labogena. After removal of SNPs with a minor allele frequency below 5%, 38 277 SNPs were retained for analysis. Simulations were carried out in nine scenarios, with heritability (h^2^) of 0.1, 0.4 or 0.7 and 10, 100 or 1000 biallelic QTL, in both the full dataset and a subset consisting of 1500 randomly selected individuals from the full dataset. Table 1 summarizes the nine simulation scenarios. For the scenarios with 100 QTL and a heritability of 0.4 or 0.7, the simulation was run 11 times. It was run only once for the others.

**Table 1:** Simulated scenarios

**Number of QTL**	**10**			**100**			**1000**		
Number of large QTL	1			5			50		
Number of medium QTL	2			20			200		
Number of small QTL	7			75			750		
**Heritability**	**0**.**1**	**0**.**4**	**0**.**7**	**0**.**1**	**0**.**4**	**0**.**7**	**0**.**1**	**0**.**4**	**0**.**7**
Variance of large QTL	2.86	11.45	20.05	0.35	1.40	2.45	0.04	0.14	0.25
Variance of medium QTL	0.95	3.82	6.68	0.12	0.47	0.82	0.01	0.05	0.08
Variance of small QTL	0.32	1.27	2.23	0.04	0.16	0.27	0.00	0.02	0.03

Number of large, medium, and small QTL, with the variance explained by each QTL; the phenotypic variance was 100 and, together, the simulated QTL explained 70% of the genetic variance.

The simulated QTL were divided into three groups: large, medium and small QTL. In the scenarios with 10 QTL, there were one large, two medium and seven small QTL, in the scenarios with 100 QTL, five large, 20 medium and 75 small QTL, and in the scenarios with 1000 QTL, 50 large, 200 medium and 750 small QTL. With the high QTL density in the scenarios with 1000 QTL, the goal was to assess whether the model could distinguish the larger QTL from the smaller ones. Together, the QTL explained 70% of the genetic variance. Large and medium QTL explained respectively 9 and 3 times the variance of a small QTL. The locations of the QTL were determined by random sampling of the SNP number. The QTL were subsequently discarded from the genotype file used in later analyses to estimate the SNP effects. The phenotypic variance $\sigma_{p}^{2}\;$ was set at 100.

Allele substitution effects (*a*_*k*_) were scaled as follows. The frequency *p*_*k*_ of one of the alleles of the *k*^*th*^ QTL was computed in order to adjust QTL effect *a*_*k*_ such that $2p_{k}\left(1-p_{k}\right)a_{k}^{2}$ equalled the part of the variance explained by the QTL. To generate residual polygenic effects, breeding values of founder animals were drawn from a normal distribution $N(0,\sigma_{u}^{2})$ with polygenic variance $\sigma_{u}^{2}$ set to 30% of the total genetic variance. Breeding values of individuals with known parents were drawn from a normal distribution $N\left(0.5\left(u_{s}+u_{d}\right),0.5\sigma_{u}^{2}\right)$ where *u*_*s*_ and *u*_*d*_ equal the breeding values of an individual’s sire and dam. Breeding values of individuals with a known sire but unknown dam were drawn from a normal distribution $N\left(0.5u_{s},0.75\sigma_{u}^{2}\right)$. The simulated performance of an individual was calculated as the sum of its polygenic value, the sum of its QTL effects and an environmental effect drawn from a normal distribution $N\left(0,\left(1-h^{2}\right)\sigma_{p}^{2}\right)$.

### QTL detection

The effect of each SNP was estimated using methods Bayes Cπ and Bayes C [13] as implemented in the GS3 software [25]. For both methods, the statistical model was:

$$y_{i}=\mu +u_{i}+\sum_{k=1}^{K}z_{\mathrm{ik}}+e_{i},$$

where *y*_*i*_ is the phenotype for individual *i*, *μ* the overall mean, *K* the number of markers, *z*_*ik*_ the genotype of individual *i* for marker *k* coded 0, 1 or 2 depending on the number of copies of a given marker allele the individual carried, *a*_*k*_ the additive effect of marker *k*, and *e*_*i*_ the random residual for individual *i*.

All unknown parameters were assigned prior distributions and sampled with a Monte Carlo Markov chain (MCMC) using Gibbs sampling. The MCMC was run for 180 000 iterations, with a burn-in of 20 000 iterations and thin interval of 50. The prior used for *a*_*k*_ was a mixture distribution that equalled:

$$\left(a_{k}\right|\pi ,\sigma_{a}^{2}\sim \left\{\begin{array}{c} 0\;\mathrm{with}\;\mathrm{probability}\;\pi ,\\ N\left(0,\sigma_{a}^{2}\right)\;\mathrm{with}\;\mathrm{probability}\;(1-\pi ), \end{array}\right)$$

Where $\sigma_{a}^{2}$ is the common marker effect variance and the hyper parameter π is the prior probability that the effect of marker *k* is 0. Variances $\sigma_{u}^{2}$, $\sigma_{a}^{2}$ and $\sigma_{e}^{2}$ were assigned inverted chi-square distributions with *v* = 4.2 degrees of freedom and scale parameter $S^{2}=\frac{{\hat{\sigma }}^{2}\left(v-2\right)}{v}$ where ${\hat{\sigma }}^{2}$ equals the prior value of $\sigma_{u}^{2},$$\sigma_{a}^{2}$ or $\sigma_{e}^{2}$. When Bayes Cπ was used, π was assigned a uniform distribution and sampled with the MCMC. For Bayes C, π was fixed at 0.99, based on the estimates obtained with Bayes Cπ in the scenarios for which π converged.

As QTL detection criterion, the posterior inclusion probability (PIP) was used, which is the proportion of iterations that included a specific marker in the model. Detection was based on either the PIP of individual markers, or the PIP summed over intervals of adjacent markers. Different interval sizes were tested, consisting of 20, 40, 60 or 80 markers, corresponding on average across the genome respectively to 1.3, 2.6, 3.9 and 5.3 Mb. Because our goal was to identify large QTL rather than all QTL, the 10 markers with the highest PIP that were more than 4 Mb apart or the 10 non-overlapping intervals with the highest PIP were declared QTL. In the simulation, a detected QTL was denoted false positive if there was no true QTL within a distance of 2 Mb on either side of the marker or within the interval, depending on the criterion that was used.

### Prediction of the status at the causative mutation

Prior to analysis, genotypes were phased in order to define haplotypes. Phasing was done using DagPhase [26], accounting for both family structure and population linkage disequilibrium. To predict the status of an individual for a simulated causative mutation, first the estimated effects of all markers in an identified QTL interval were summed up for each haplotype that the individual carried. Second, the absolute value of the difference between the estimated effects of an individual’s two haplotypes was used to group individuals by partitioning around medoids (PAM) [27], as implemented in the fpc R-package [28], where the number of clusters (*n*) is estimated on the basis of optimum average silhouette [29]. The PAM algorithm was as follows:

1. *n* medoids were randomly selected from the data.

2. All non-medoids were assigned to the closest medoid and the costs of configurations, when the medoids and data points were switched, were calculated.

3. The configuration with the lowest cost was selected.

4. Steps 2 and 3 were repeated until there was no longer any change in the medoids.

A range of 2 to 4 was used to estimate *n*. The status of animals in the cluster with the lowest haplotype difference was denoted homozygous, and that of animals in the cluster with the highest difference was denoted heterozygous. If more than two clusters were present, the status of animals in the other clusters was denoted unknown. To evaluate the accuracy of status prediction, predicted statuses were compared with the true statuses in order to compute the proportion of correct, incorrect and unknown statuses.

## Results

### Posterior distribution of π

**Figure 1:**
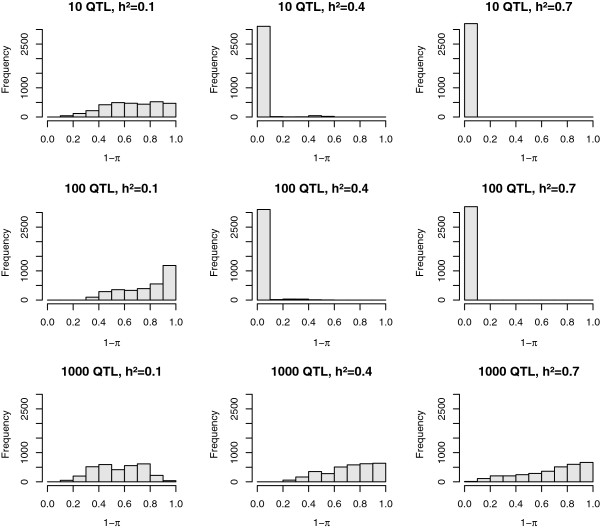
**Posterior distribution of the proportion of markers included in Bayes Cπ** (**1**-**π**) **for simulated scenarios.** The MCMC chain was run for 180 000 iterations; scenarios differed in the number of QTL (10, 100, or 1000) and heritability (h^2^ = 0.1, 0.4, or 0.7).

**Figure 2:**
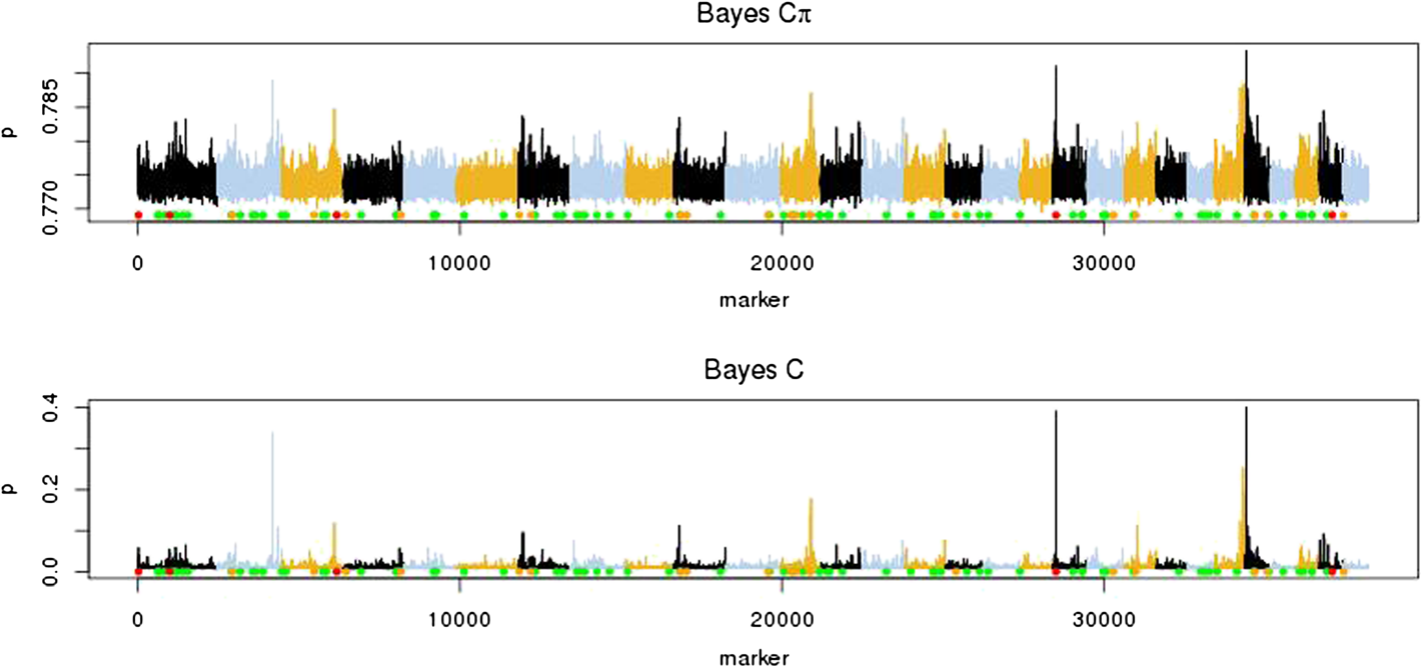
**Posterior inclusion probability** (**p**) **across the genome for Bayes Cπ and Bayes C.** Scenario with 100 simulated QTL and heritability equal to 0.1; the red, orange and green dots on the x-axes show the true positions of the large, medium, and small simulated QTL, respectively.

The posterior distribution of the proportion of markers included in the Bayes Cπ model (1-π) for the full dataset is shown in Figure 1. Only in the scenarios with 10 or 100 QTL and a heritability of 0.4 or 0.7, did the posterior distribution of π show a strong peak. In the other scenarios, values taken by π ranged from 0 to 1. In the subset data, a clear peak in the posterior distribution of π was only observed with a heritability of 0.7 and 10 or 100 QTL. For scenarios without a clear peak, the average PIP was much higher than for scenarios with a strong peak. As shown in Figure 2, for a scenario without a strong peak, the average PIP was high and the peaks were much smaller and broader for Bayes Cπ than for Bayes C. Table 2 shows the average number of markers included in the model in the full dataset. For all scenarios, the number of included markers was much higher than the number of simulated QTL.

**Table 2:** Posterior means of the number of markers included in Bayes Cπ

**Number of QTL simulated**	**Heritability**					
	**Full data**			**Subset**		
	**0**.**1**	**0**.**4**	**0**.**7**	**0**.**1**	**0**.**4**	**0**.**7**
10	25 173	503	43	28 633	24 782	1374
100	29 504	743	418	17 663	27 072	11 906
1000	20 698	26 612	25 249	19 681	21 766	26 895

### Influence of heritability and number of records

The accuracy of QTL detection was influenced by both heritability and the number of simulated QTL. Table 3 shows the numbers of correctly identified QTL and false positives for the nine scenarios, both when intervals consisting of 60 subsequent markers were selected on the basis of the sum of the PIP of all markers in the interval, and when markers were individually selected according to their PIP. Both for selection of intervals and of markers, the number of correctly identified QTL and the number of false positives were highly affected by heritability and number of QTL. The number of false positives decreased with increasing heritability. Furthermore, the number of false positives was higher with 100 QTL than with 10 QTL. With 1000 QTL, due to the large number of QTL, there was on average 1.6 QTL present per haplotype region and thus hardly any false positives were found. Most detected QTL were, however, small, meaning that most large QTL were not in the 10 selected regions.

**Table 3:** Correctly identified QTL and false positives using Bayes C

**Size and number of simulated QTL**		**Heritability**					
		**Selection of intervals**			**Selection of markers**		
		**0**.**1**	**0**.**4**	**0**.**7**	**0**.**1**	**0**.**4**	**0**.**7**
Large	1	1	1	1	1	1	1
Medium	2	0	2	2	0	1	1
Small	7	4	4	5	3	5	7
False positives		5	3	2	6	3	1
Large	5	1	2	4	1	2	4
Medium	20	1	6	6	2	2	4
Small	75	1	2	2	1	1	2
False positives		7	3	2	7	5	2
Large	50	0	1	1	1	4	5
Medium	200	7	9	8	5	7	5
Small	750	8	16	16	14	16	18
False positives		1	0	0	1	0	1

Number of correctly identified QTL categorised by size of the QTL variance (large, medium or small), and number of false positives, with QTL detection based on the posterior inclusion probability summed up over intervals of 60 adjacent markers or the posterior inclusion probability of individual markers; the 10 best intervals or markers were denoted as QTL; more than one QTL can be present in an interval, so that the total number of QTL identified can be larger than 10.

In most cases, selection of intervals resulted in fewer or equal false positives than selection of markers, except in the scenario with 10 QTL and a heritability of 0.7, which had two and one false positives for selection based on intervals and markers, respectively.

Generally, the proportion of large QTL detected was greater than the proportions of medium and small QTL detected. However, with 1000 QTL, hardly any large QTL were detected when intervals were selected but larger QTL were detected when selecting individual markers. For example, with 1000 QTL and a heritability of 0.7, selecting intervals led to the detection of only one large QTL, while selection of markers detected five large QTL.

For the scenarios with 100 QTL and a heritability of 0.4 and 0.7, the simulation was run ten times. The results of these repeated simulations are shown in Table 4. The mean number of false positives over ten runs was 0.40 and 0.10 for heritabilities of 0.4 and 0.7, respectively.

**Table 4:** Correctly identified QTL and false positives using Bayes C over 10 replicates

	**Heritability** = **0**.**4**				**Heritability** = **0**.**7**			
	**Large**	**Medium**	**Small**	**False**	**Large**	**Medium**	**Small**	**False**
Mean	3.70	4.5	3.60	0.40	2	5.4	8.5	0.1
Min	3	3	2	0	0	3	6	0
Max	4	8	6	2	3	8	12	1
SD	0.48	1.78	1.51	0.70	0.94	1.90	2.55	0.32

Mean, minimum, maximum and standard deviation of the number of correctly identified QTL categorised by effect size (large, medium or small), and number of false positives, based on 10 replicates; QTL detection was based on the posterior inclusion probability summed up over intervals of 60 adjacent markers; the best 10 intervals were denoted as QTL; 100 QTL were simulated, of which 5 were large, 20 medium and 75 small.

### Number of records, interval size and number of detected QTL

**Figure 3:**
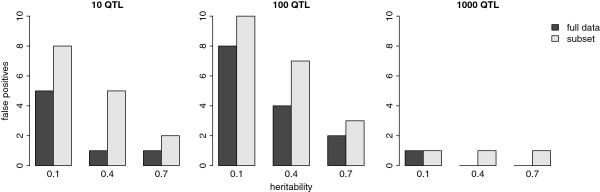
**Number of false positives after selecting 10 intervals with the highest summed posterior inclusion probabilities.** Intervals consisted of 60 adjacent markers; the full dataset and the subset consisted of respectively 4387 and 1500 records.

The number of records had a strong influence on the accuracy of QTL detection. Figure 3 shows the number of false positives for both the full dataset and the subset. The number of false positives was higher for the subset than for the full dataset. The difference was largest in the scenarios with 10 QTL and a heritability of 0.4, which had only one false positive with the full dataset but five false positives with the subset.

**Figure 4:**
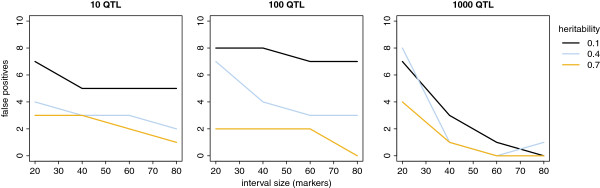
**Number of false positives as a function of interval size.** The ten intervals with the highest summed posterior inclusion probabilities were selected.

Figure 4 shows the effect of interval size on the number of false positives. In the scenarios with 1000 QTL, the genome was saturated with QTL, and there were hardly any intervals without a QTL. As we were interested in identifying the largest QTL, we redefined the number of false positives as the number of detected intervals without QTL or with only small QTL for these scenarios. The number of false positives decreased with increasing interval size. This decrease was stronger when the number of QTL was greater, and was most pronounced with a heritability of 0.4.

**Figure 5:**
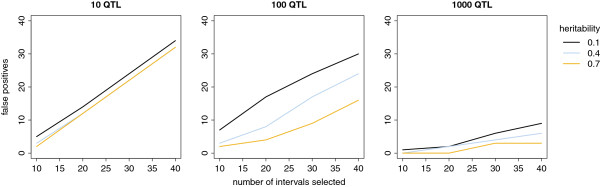
**Number of false positives according to the number of selected intervals.** Intervals consisted of 60 adjacent markers.

Figure 5 shows the number of false positives when more than 10 intervals were selected. The number of false positives in the scenarios with 1000 QTL was defined as previously described. In all scenarios, the number of false positives increased when more intervals were selected. This increase was lower with a higher heritability, especially with 100 QTL.

### Prediction of status at the causative mutation

**Figure 6:**
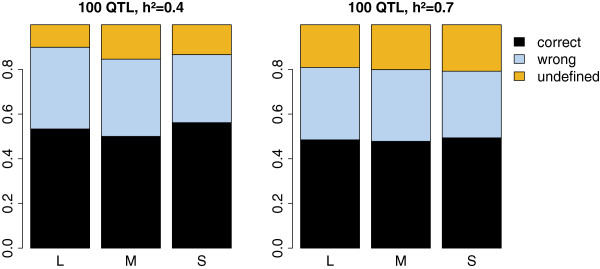
**Proportion of correctly and incorrectly predicted and undefined statuses at the detected QTL.** For scenarios with heritabilities (h^2^) of 0.4 and 0.7, 100 QTL were simulated 10 times, of which five were large (L), 20 medium (M) and 75 small (S).

**Figure 7:**
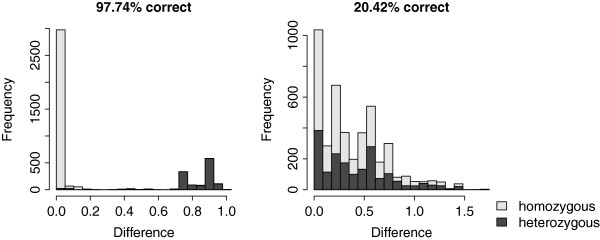
**Distribution of absolute difference between estimated haplotype effects in an identified QTL region.** Haplotype effects were estimated by the sum of the estimated marker effects for all markers in the haplotype; distribution for the QTL with the highest (left) and lowest (right) status prediction accuracy.

**Figure 8:**
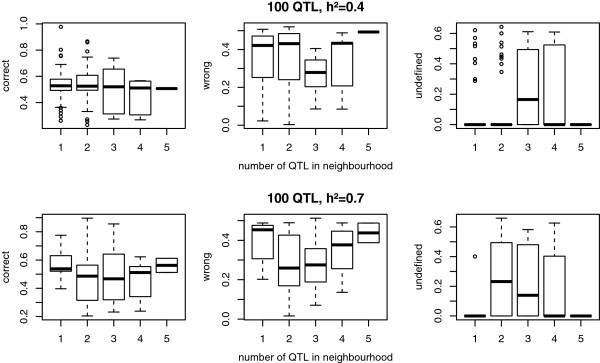
**Effect of the number of QTL on accuracy of status prediction for the causative mutation.** Box plots showing the proportion of correctly and incorrectly predicted and undefined statuses, for scenarios with heritabilities (h^2^) of 0.4 and 0.7; in both scenarios, 100 QTL were simulated 10 times, of which five were large (L), 20 medium (M) and 75 small (S); the x-axis shows the number of QTL within a distance of 10 Mb of one another.

Figure 6 shows the proportion of correct, incorrect and undefined statuses for the scenarios with 100 QTL and a heritability of 0.4 and 0.7. On average, QTL status prediction was correct for approximately 50% of the individuals but this percentage differed strongly between QTL, ranging from 20.4% to 97.7%. Size of the QTL did not affect the percentage of correctly predicted statuses. The shape of the distributions of the haplotype differences varied largely between QTL. As shown in Figure 7, for a QTL with status predicted with almost 100% accuracy, the homozygous and heterozygous individuals formed two clearly separated groups, while for the QTL that gave the poorest status predictions, there was complete overlap between homozygous and heterozygous individuals. The accuracy of status prediction was affected by the number of QTL in the neighbourhood of the causative mutation. Figure 8 shows that the proportion of correctly predicted statuses was highest when there was no other QTL within 10 Mb of the causative mutation, although this effect was larger with a heritability of 0.4 than with a heritability of 0.7. Furthermore, when there was only one QTL present, clustering almost always resulted in two groups and no unknowns, while with more QTL close to one another, the individuals were divided into three or four groups, so that status was undefined for some animals.

## Discussion

The accuracy of QTL mapping was affected by heritability, number of QTL, and number of records. In the subset data, the number of false positives was very high in all scenarios, demonstrating the need for a large dataset. With a low heritability, the QTL effects were smaller than with a higher heritability, making it more difficult to accurately locate the QTL and thus resulting in a greater number of false positives. Therefore, better results will be obtained when progeny-based evaluations are used rather than individual phenotypes, since the heritability of progeny means is higher. This strategy based on daughter-yield-deviations or deregressed estimated breeding values is quite frequently used in dairy cattle [30].

When the number of QTL to explain a given amount of genetic variance increases, the individual QTL effects become smaller and the QTL thus become more difficult to detect and locate. Consequently, the number of false positives increased when the number of QTL increased from 10 to 100. However, with a further increase to 1000 QTL, the number of false positives decreased to 1 or 0 because with 1000 QTL on a total of 37 227 markers, an interval of 60 markers includes on average 1.6 QTL. Randomly selecting intervals would give the same result. The motivation for QTL mapping is the identification of large rather than small QTL. In the scenarios with 1000 QTL, mainly small QTL were found. It is, however, important to realise that large QTL in the scenarios with 1000 QTL had about the same size as the small QTL in the scenarios with 100 QTL and were much smaller than the small QTL in the scenarios with 10 QTL (Table 1). For example, with a heritability of 0.4, a large QTL in the scenario with 1000 QTL explains 0.14% of the total phenotypic variance, while small QTL in the scenarios with 100 and 10 QTL explain 0.16% and 1.27%, respectively. The small size of the large QTL in the scenarios with 1000 QTL clearly makes it difficult to detect these QTL. Furthermore, with the large number of small and medium QTL in these scenarios, several small and medium QTL together can have a larger effect than a single large QTL. Based on our results, the variance explained by the QTL in an interval should roughly explain at least 1% of the phenotypic variance in order for the QTL to be detected.

QTL detection can be based on several criteria, including marker effect, variance and PIP, either for each marker individually or summed up over an interval of adjacent markers. In this study, PIP was used as the detection criterion, for both markers and intervals. If several markers are in linkage disequilibrium with a QTL, the QTL effect can spread over several markers. In that case, selection based on intervals can give more accurate results than selection based on individual markers [10]. In most scenarios evaluated here, fewer false positives were found when intervals were selected than when markers were selected. However, as shown in Table 3, selection of intervals resulted in the detection of mainly small QTL and hardly any large QTL in the scenarios with 1000 QTL, while there were 50 large QTL simulated and only the best 10 intervals were selected. In contrast, when detection was based on individual markers, a much larger proportion of the detected QTL were large QTL. The intervals that were selected in this case were generally located in the proximity of several medium or large QTL. Selection of intervals can thus result in the identification of regions that are close to the largest number of QTL rather than regions that include the largest QTL. Therefore, for polygenic traits, selection of markers can give better results, in terms of identifying large QTL, than selection of intervals. Since the number of QTL influencing a trait is generally unknown, it is difficult to decide which approach to use, and the best option could be to combine both approaches and declare only QTL that have a high ranking for both criteria.

When the interval size increased, the number of false positives decreased, as shown in Figure 4. However, increasing interval size makes the subsequent search for causative mutations more difficult, since the search area increases as well. Based on our results, interval sizes containing 40 to 60 markers and corresponding to on average 2.6 to 3.9 Mb, seem to be a reasonable compromise for the usual designs currently available in animal breeding.

In results reported here, the 10 best markers or intervals were selected and denoted as QTL, which is only a small fraction of the total number of QTL in the scenarios with 100 and 1000 QTL. However, when more QTL are selected, the number of false positives increases rapidly. This is in agreement with other studies, in which Bayes A and B [9] and Bayes Cπ [14] were successful in locating the large QTL but missed the smaller ones. The Bayesian genomic selection models are thus suitable for QTL mapping when the aim is to find the major QTL.

In addition to previously discussed factors, linkage disequilibrium between markers and QTL is required for the markers to pick up the effect of a QTL. The accuracy of genomic prediction with Bayesian models is lower in populations with a large effective population size and thus a low amount of linkage disequilibrium [4]. In this study, data were simulated using genotypes of French Holstein cattle. When applied to populations with a different effective population size, the accuracy of QTL mapping is expected to be different than observed here. In contrast to genomic prediction, the motivation for QTL mapping is not to capture the maximum amount of information on the trait but rather to capture information on markers close to the causative mutation. In a population in which linkage disequilibrium is present only over short distances, only markers near the causative mutation will have an effect, and thus a more accurate estimate of the QTL location is expected than with a population with long range linkage disequilibrium.

The posterior distribution of π was affected by heritability, number of QTL and number of records. In scenarios for which the posterior distribution of π lacked a clear peak were the scenarios with a low heritability or a large number of QTL. In these scenarios, the variance explained by an individual QTL was small, increasing the difficulty of locating the QTL. For scenarios with a clear peak, the number of SNPs included in the model was much larger than the number of simulated QTL. This agrees with results of Habier et al. [13] who reported that the number of SNPs with effect estimated on the basis of π was overestimated. In their results, the number of markers included in the model increased with decreasing heritability. The accuracy of breeding value predictions from Bayesian genomic selection models decreases and becomes more similar to the accuracy of GBLUP with decreasing heritability, increasing number of QTL and decreasing number of records, [4,20-23]. The GBLUP model uses all markers to estimate breeding values on the basis of the genomic relationship between animals rather than based on individual marker effects [31]. In scenarios that did not show a clear peak in the posterior distribution of π, individual marker effects were small and thus more difficult to estimate. A possible explanation for the lack of a clear peak could be that in these cases, Bayes Cπ acts similar to the GBLUP model and relies more on the genomic relationship between animals than on QTL effects, and therefore includes a large and variable proportion of markers in the model to capture relationships between animals. As a result, the average PIP of markers was high, and the peaks of PIP were relatively small deviations from this mean. In addition, a strong negative correlation was observed between π and $\sigma_{a}^{2}$. When π was fixed, the deviations from the mean were larger and sharper, as in Figure 2, making it easier to locate QTL.

The accuracy of the predicted statuses of the causative mutations differed strongly between QTL. For some QTL, the accuracy was almost 100%, while for others the status of almost none of the individuals was correctly predicted. The accuracy of status predictions was affected by the presence of other QTL nearby. When the effect of the haplotypes is influenced by several QTL rather than by only one, the status of the QTL can be predicted correctly based on the difference between haplotype effects only if the statuses of all QTL influencing the haplotype effect are in concordance with each other. When this is not the case, the differences between haplotype effects of homozygous and heterozygous individuals overlap and it is not possible to cluster the individuals in two groups. Although the prediction of QTL status was influenced by the presence of other QTL nearby, their presence seemed to explain only part of the variation in prediction accuracy between QTL. It is not clear what caused the rest of the variation.

In order to identify the causative mutation, it is important that the predicted QTL status of individuals is correct. Otherwise the true polymorphism could be eliminated because of lack of concordance with the QTL status. Although the status prediction was far from perfect, histograms of the haplotype differences clearly demonstrated whether the clustering was successful or not. As shown in Figure 7, when two clearly separated groups were visible, almost 100% of the statuses were correctly predicted, while histograms did not show separate groups for QTL with low prediction accuracy.

## Conclusions

The performance of Bayes Cπ and Bayes C was highly affected by heritability, number of QTL and number of records. The number of false positives was high with low heritability, and decreased with increasing heritability. In polygenic scenarios, larger QTL were detected when detection was based on the PIP of individual markers than when based on the PIP summed up over a group of adjacent markers. Thus, a first conclusion is that Bayes C is a suitable method for QTL mapping when it is applied to traits with moderate to high heritability and when a large number of records are available.

For Bayes Cπ, there was no strong peak in the posterior distribution of π when heritability was low or the number of QTL was large, and a large number of SNPs were selected. In such cases, localisation of the QTL was more accurate with Bayes C. Furthermore, with moderate heritability, a large number of records was needed for π to show a clear peak in the posterior distribution. Therefore, a second conclusion is to recommend a high fixed value for π.

The accuracy of the predicted status of the causative mutation is affected by the size of the QTL, as well as by the presence of other QTL nearby. Although the accuracy in general is not very high, a histogram of the haplotype differences can be used to decide whether the clustering is correct or not and thus usable for further analysis.

## Competing interests

The authors declare that they have no competing interests.

## Authors’ contributions

IB and DB designed and carried out the study, and drafted the manuscript. SF generated and provided phased data. All authors read and approved the final manuscript.
